# Supplementary material for: Context-specific emergence and growth of the SARS-CoV-2 Delta variant
Source: Res Sq. 2021 Dec 20:rs.3.rs-1159614. Preprint. [Version 1] doi: 10.21203/rs.3.rs-1159614/v1 (PMC8722606; doi:10.21203/rs.3.rs-1159614/v1)
Supplement: 1 [file NIHPPrs1159614v1-supplement-1.pdf]

Supplementary Information

Supplementary Table 1: The COVID-19 Genomics UK (COG-UK) consortium, June 2021 V.1

The COVID-19 Genomics UK (COG-UK) consortium
June 2021 V.1

Funding acquisition, Leadership and supervision, Metadata curation, Project administration, Samples and logistics, Sequencing and analysis, Software and analysis tools, and Visualisation:
Samuel C Robson [13, 84]

Funding acquisition, Leadership and supervision, Metadata curation, Project administration, Samples and logistics, Sequencing and analysis, and Software and analysis tools:
Thomas R Connor [11, 74] and Nicholas J Loman [43]

Leadership and supervision, Metadata curation, Project administration, Samples and logistics, Sequencing and analysis, Software and analysis tools, and Visualisation:
Tanya Golubchik [5]

Funding acquisition, Leadership and supervision, Metadata curation, Samples and logistics, Sequencing and analysis, and Visualisation:
Rocio T Martinez Nunez [46]

Funding acquisition, Leadership and supervision, Project administration, Samples and logistics, Sequencing and analysis, and Software and analysis tools:
David Bonsall [5]

Funding acquisition, Leadership and supervision, Project administration, Sequencing and analysis, Software and analysis tools, and Visualisation:
Andrew Rambaut [104]

Funding acquisition, Metadata curation, Project administration, Samples and logistics, Sequencing and analysis, and Software and analysis tools:
Luke B Snell [12]

Leadership and supervision, Metadata curation, Project administration, Samples and logistics, Software and analysis tools, and Visualisation:
Rich Livett [116]

Funding acquisition, Leadership and supervision, Metadata curation, Project administration, and Samples and logistics:
Catherine Ludden [20, 70]

Funding acquisition, Leadership and supervision, Metadata curation, Samples and logistics, and Sequencing and analysis:
Sally Corden [74] and Eleni Nastouli [96, 95, 30]

Funding acquisition, Leadership and supervision, Metadata curation, Sequencing and analysis, and Software and analysis tools:
Gaia Nebbia [12]

Funding acquisition, Leadership and supervision, Project administration, Samples and logistics, and Sequencing and analysis:
Ian Johnston [116]

Leadership and supervision, Metadata curation, Project administration, Samples and logistics, and Sequencing and analysis:
Katrina Lythgoe [5], M. Estee Torok [19, 20] and Ian G Goodfellow [24]
Leadership and supervision, Metadata curation, Project administration, Samples and logistics, and Visualisation:
Jacqui A Prieto [97, 82] and Kordo Saeed [97, 83]

Leadership and supervision, Metadata curation, Project administration, Sequencing and analysis, and Software and analysis tools:
David K Jackson [116]

Leadership and supervision, Metadata curation, Samples and logistics, Sequencing and analysis, and Visualisation:
Catherine Houlihan [96, 94]

Leadership and supervision, Metadata curation, Sequencing and analysis, Software and analysis tools, and Visualisation:
Dan Frampton [94, 95]

Metadata curation, Project administration, Samples and logistics, Sequencing and analysis, and Software and analysis tools:
William L Hamilton [19] and Adam A Witney [41]

Funding acquisition, Samples and logistics, Sequencing and analysis, and Visualisation:
Giselda Bucca [101]

Funding acquisition, Leadership and supervision, Metadata curation, and Project administration:
Cassie F Pope [40, 41]

Funding acquisition, Leadership and supervision, Metadata curation, and Samples and logistics:
Catherine Moore [74]

Funding acquisition, Leadership and supervision, Metadata curation, and Sequencing and analysis:
Emma C Thomson [53]

Funding acquisition, Leadership and supervision, Project administration, and Samples and logistics:
Ewan M Harrison [116, 102]

Funding acquisition, Leadership and supervision, Sequencing and analysis, and Visualisation:
Colin P Smith [101]

Leadership and supervision, Metadata curation, Project administration, and Sequencing and analysis:
Fiona Rogan [77]

Leadership and supervision, Metadata curation, Project administration, and Samples and logistics:
Shaun M Beckwith [6], Abigail Murray [6], Dawn Singleton [6], Kirstine Eastick [37], Liz A Sheridan [98], Paul Randell [99], Leigh M Jackson [105], Cristina V Ariani [116] and Sónia Gonçalves [116]

Leadership and supervision, Metadata curation, Samples and logistics, and Sequencing and analysis:
Derek J Fairley [3, 77], Matthew W Loose [18] and Joanne Watkins [74]

Leadership and supervision, Metadata curation, Samples and logistics, and Visualisation:
Samuel Moses [25, 106]

Leadership and supervision, Metadata curation, Sequencing and analysis, and Software and analysis tools:
Sam Nicholls [43], Matthew Bull [74] and Roberto Amato [116]

Leadership and supervision, Project administration, Samples and logistics, and Sequencing and analysis:
Darren L Smith [36, 65, 66]

Leadership and supervision, Sequencing and analysis, Software and analysis tools, and Visualisation:
David M Aanensen [14, 116] and Jeffrey C Barrett [116]

Metadata curation, Project administration, Samples and logistics, and Sequencing and analysis:
Dinesh Aggarwal [20, 116, 70], James G Shepherd [53], Martin D Curran [71] and Surendra Parmar [71]

Metadata curation, Project administration, Sequencing and analysis, and Software and analysis tools:
Matthew D Parker [109]

Metadata curation, Samples and logistics, Sequencing and analysis, and Software and analysis tools:
Catryn Williams [74]

Metadata curation, Samples and logistics, Sequencing and analysis, and Visualisation:
Sharon Glaysher [68]

Metadata curation, Sequencing and analysis, Software and analysis tools, and Visualisation:
Anthony P Underwood [14, 116], Matthew Bashton [36, 65], Nicole Pacchiarini [74], Katie F Loveson [84] and Matthew Byott [95, 96]

Project administration, Sequencing and analysis, Software and analysis tools, and Visualisation:
Alessandro M Carabelli [20]

Funding acquisition, Leadership and supervision, and Metadata curation:
Kate E Templeton [56, 104]

Funding acquisition, Leadership and supervision, and Project administration:
Thushan I de Silva [109], Dennis Wang [109], Cordelia F Langford [116] and John Sillitoe [116]

Funding acquisition, Leadership and supervision, and Samples and logistics:
Rory N Gunson [55]

Funding acquisition, Leadership and supervision, and Sequencing and analysis:
Simon Cottrell [74], Justin O'Grady [75, 103] and Dominic Kwiatkowski [116, 108]

Leadership and supervision, Metadata curation, and Project administration:
Patrick J Lillie [37]

Leadership and supervision, Metadata curation, and Samples and logistics:
Nicholas Cortes [33], Nathan Moore [33], Claire Thomas [33], Phillipa J Burns [37], Tabitha W Mahungu [80] and Steven Liggett [86]

Leadership and supervision, Metadata curation, and Sequencing and analysis:
Angela H Beckett [13, 81] and Matthew TG Holden [73]

Leadership and supervision, Project administration, and Samples and logistics:
Lisa J Levett [34], Husam Osman [70, 35] and Mohammed O Hassan-Ibrahim [99]

Leadership and supervision, Project administration, and Sequencing and analysis:
David A Simpson [77]

Leadership and supervision, Samples and logistics, and Sequencing and analysis:
Meera Chand [72], Ravi K Gupta [102], Alistair C Darby [107] and Steve Paterson [107]

Leadership and supervision, Sequencing and analysis, and Software and analysis tools:
Oliver G Pybus [23], Erik M Volz [39], Daniela de Angelis [52], David L Robertson [53], Andrew J Page [75] and Inigo Martincorena [116]

Leadership and supervision, Sequencing and analysis, and Visualisation:
Louise Aigrain [116] and Andrew R Bassett [116]

Metadata curation, Project administration, and Samples and logistics:
Nick Wong [50], Yusri Taha [89], Michelle J Erkiert [99] and Michael H Spencer Chapman [116, 102]

Metadata curation, Project administration, and Sequencing and analysis:
Rebecca Dewar [56] and Martin P McHugh [56, 111]

Metadata curation, Project administration, and Software and analysis tools:
Siddharth Mookerjee [38, 57]

Metadata curation, Project administration, and Visualisation:
Stephen Aplin [97], Matthew Harvey [97], Thea Sass [97], Helen Umpleby [97] and Helen Wheeler [97]

Metadata curation, Samples and logistics, and Sequencing and analysis:
James P McKenna [3], Ben Warne [9], Joshua F Taylor [22], Yasmin Chaudhry [24], Rhys Izuagbe [24], Aminu S Jahun [24], Gregory R Young [36, 65], Claire McMurray [43], Clare M McCann [65, 66], Andrew Nelson [65, 66] and Scott Elliott [68]

Metadata curation, Samples and logistics, and Visualisation:
Hannah Lowe [25]

Metadata curation, Sequencing and analysis, and Software and analysis tools:
Anna Price [11], Matthew R Crown [65], Sara Rey [74], Sunando Roy [96] and Ben Temperton [105]

Metadata curation, Sequencing and analysis, and Visualisation:
Sharif Shaaban [73] and Andrew R Hesketh [101]

Project administration, Samples and logistics, and Sequencing and analysis:
Kenneth G Laing [41], Irene M Monahan [41] and Judith Heaney [95, 96, 34]

Project administration, Samples and logistics, and Visualisation:
Emanuela Pelosi [97], Siona Silviera [97] and Eleri Wilson-Davies [97]

Samples and logistics, Software and analysis tools, and Visualisation:
Helen Fryer [5]

Sequencing and analysis, Software and analysis tools, and Visualization:

Helen Adams [4], Louis du Plessis [23], Rob Johnson [39], William T Harvey [53, 42], Joseph Hughes [53], Richard J Orton [53], Lewis G Spurgin [59], Yann Bourgeois [81], Chris Ruis [102], Áine O'Toole [104], Marina Gourtovaia [116] and Theo Sanderson [116]

Funding acquisition, and Leadership and supervision:
Christophe Fraser [5], Jonathan Edgeworth [12], Judith Breuer [96, 29], Stephen L Michell [105] and John A Todd [115]

Funding acquisition, and Project administration:
Michaela John [10] and David Buck [115]

Leadership and supervision, and Metadata curation:
Kavitha Gajee [37] and Gemma L Kay [75]

Leadership and supervision, and Project administration:
Sharon J Peacock [20, 70] and David Heyburn [74]

Leadership and supervision, and Samples and logistics:
Katie Kitchman [37], Alan McNally [43, 93], David T Pritchard [50], Samir Dervisevic [58], Peter Muir [70], Esther Robinson [70, 35], Barry B Vipond [70], Newara A Ramadan [78], Christopher Jeanes [90], Danni Weldon [116], Jana Catalan [118] and Neil Jones [118]

Leadership and supervision, and Sequencing and analysis:
Ana da Silva Filipe [53], Chris Williams [74], Marc Fuchs [77], Julia Miskelly [77], Aaron R Jeffries [105], Karen Oliver [116] and Naomi R Park [116]

Metadata curation, and Samples and logistics:
Amy Ash [1], Cherian Koshy [1], Magdalena Barrow [7], Sarah L Buchan [7], Anna Mantzouratou [7], Gemma Clark [15], Christopher W Holmes [16], Sharon Campbell [17], Thomas Davis [21], Ngee Keong Tan [22], Julianne R Brown [29], Kathryn A Harris [29, 2], Stephen P Kidd [33], Paul R Grant [34], Li Xu-McCrae [35], Alison Cox [38, 63], Pinglawathee Madona [38, 63], Marcus Pond [38, 63], Paul A Randell [38, 63], Karen T Withell [48], Cheryl Williams [51], Clive Graham [60], Rebecca Denton-Smith [62], Emma Swindells [62], Robyn Turnbull [62], Tim J Sloan [67], Andrew Bosworth [70, 35], Stephanie Hutchings [70], Hannah M Pymont [70], Anna Casey [76], Liz Ratcliffe [76], Christopher R Jones [79, 105], Bridget A Knight [79, 105], Tanzina Haque [80], Jennifer Hart [80], Dianne Irish-Tavares [80], Eric Witele [80], Craig Mower [86], Louisa K Watson [86], Jennifer Collins [89], Gary Eltringham [89], Dorian Crudgington [98], Ben Macklin [98], Miren Iturriza-Gomara [107], Anita O Lucaci [107] and Patrick C McClure [113]

Metadata curation, and Sequencing and analysis:
Matthew Carlile [18], Nadine Holmes [18], Christopher Moore [18], Nathaniel Storey [29], Stefan Rooke [73], Gonzalo Yebra [73], Noel Craine [74], Malorie Perry [74], Nabil-Fareed Alikhan [75], Stephen Bridgett [77], Kate F Cook [84], Christopher Fearn [84], Salman Goudarzi [84], Ronan A Lyons [88], Thomas Williams [104], Sam T Haldenby [107], Jillian Durham [116] and Steven Leonard [116]

Metadata curation, and Software and analysis tools:
Robert M Davies [116]

Project administration, and Samples and logistics:
Rahul Batra [12], Beth Blane [20], Moira J Spyer [30, 95, 96], Perminder Smith [32, 112], Mehmet Yavus [85, 109], Rachel J Williams [96], Adhyana IK Mahanama [97], Buddhini Samaraweera [97], Sophia T Girgis [102], Samantha E Hansford [109], Angie Green [115], Charlotte Beaver [116], Katherine L Bellis [116, 102], Matthew J Dorman [116], Sally Kay [116], Liam Prestwood [116] and Shavanthi Rajatileka [116]

Project administration, and Sequencing and analysis:
Joshua Quick [43]

Project administration, and Software and analysis tools:
Radoslaw Poplawski [43]

Samples and logistics, and Sequencing and analysis:
Nicola Reynolds [8], Andrew Mack [11], Arthur Morriss [11], Thomas Whalley [11], Bindi Patel [12], Iliana Georgana [24], Myra Hosmillo [24], Malte L Pinckert [24], Joanne Stockton [43], John H Henderson [65], Amy Hollis [65], William Stanley [65], Wen C Yew [65], Richard Myers [72], Alicia Thornton [72], Alexander Adams [74], Tara Annett [74], Hibo Asad [74], Alec Birchley [74], Jason Coombes [74], Johnathan M Evans [74], Laia Fina [74], Bree Gatica-Wilcox [74], Lauren Gilbert [74], Lee Graham [74], Jessica Hey [74], Ember Hilvers [74], Sophie Jones [74], Hannah Jones [74], Sara Kumziene-Summerhayes [74], Caoimhe McKerr [74], Jessica Powell [74], Georgia Pugh [74], Sarah Taylor [74], Alexander J Trotter [75], Charlotte A Williams [96], Leanne M Kermack [102], Benjamin H Foulkes [109], Marta Gallis [109], Hailey R Hornsby [109], Stavroula F Louka [109], Manoj Pohare [109], Paige Wolverson [109], Peijun Zhang [109], George MacIntyre-Cockett [115], Amy Trebes [115], Robin J Moll [116], Lynne Ferguson [117], Emily J Goldstein [117], Alasdair Maclean [117] and Rachael Tomb [117]

Samples and logistics, and Software and analysis tools:
Igor Starinskij [53]

Sequencing and analysis, and Software and analysis tools:
Laura Thomson [5], Joel Southgate [11, 74], Moritz UG Kraemer [23], Jayna Raghwani [23], Alex E Zarebski [23], Olivia Boyd [39], Lily Geidelberg [39], Chris J Illingworth [52], Chris Jackson [52], David Pascall [52], Sreenu Vattipally [53], Timothy M Freeman [109], Sharon N Hsu [109], Benjamin B Lindsey [109], Keith James [116], Kevin Lewis [116], Gerry Tonkin-Hill [116] and Jaime M Tovar-Corona [116]

Sequencing and analysis, and Visualisation:
MacGregor Cox [20]

Software and analysis tools, and Visualisation:
Khalil Abudahab [14, 116], Mirko Menegazzo [14], Ben EW Taylor MEng [14, 116], Corin A Yeats [14], Afrida Mukaddas [53], Derek W Wright [53], Leonardo de Oliveira Martins [75], Rachel Colquhoun [104], Verity Hill [104], Ben Jackson [104], JT McCrone [104], Nathan Medd [104], Emily Scher [104] and Jon-Paul Keatley [116]

Leadership and supervision:
Tanya Curran [3], Sian Morgan [10], Patrick Maxwell [20], Ken Smith [20], Sahar Eldirdiri [21], Anita Kenyon [21], Alison H Holmes [38, 57], James R Price [38, 57], Tim Wyatt [69], Alison E Mather [75], Timofey Skvortsov [77] and John A Hartley [96]

Metadata curation:
Martyn Guest [11], Christine Kitchen [11], Ian Merrick [11], Robert Munn [11], Beatrice Bertolusso [33], Jessica Lynch [33], Gabrielle Vernet [33], Stuart Kirk [34], Elizabeth Wastnedge [56], Rachael Stanley [58], Giles Idle [64], Declan T Bradley [69, 77], Jennifer Poyner [79] and Matilde Mori [110]

Project administration:
Owen Jones [11], Victoria Wright [18], Ellena Brooks [20], Carol M Churcher [20], Mireille Fragakis [20], Katerina Galai [20, 70], Andrew Jermy [20], Sarah Judges [20], Georgina M McManus [20], Kim S Smith [20], Elaine Westwick [20], Stephen W Attwood [23], Frances Bolt [38, 57], Alisha Davies [74], Elen De Lacy [74], Fatima Downing [74], Sue Edwards [74], Lizzie Meadows [75], Sarah Jeremiah [97], Nikki Smith [109] and Luke Foulser [116]

Samples and logistics:
Themoula Charalampous [12, 46], Amita Patel [12], Louise Berry [15], Tim Boswell [15], Vicki M Fleming [15], Hannah C Howson-Wells [15], Amelia Joseph [15], Manjinder Khakh [15], Michelle M Lister [15], Paul W Bird [16], Karlie Fallon [16], Thomas Helmer [16], Claire L McMurray [16], Mina Odedra [16], Jessica Shaw [16], Julian W Tang [16], Nicholas J Willford [16], Victoria Blakey [17], Veena Raviprakash [17], Nicola Sheriff [17], Lesley-Anne Williams [17], Theresa Feltwell [20], Luke Bedford [26], James S Cargill [27], Warwick Hughes [27], Jonathan Moore [28], Susanne Stonehouse [28], Laura Atkinson [29], Jack CD Lee [29], Dr Divya Shah [29], Adela Alcolea-Medina [32, 112], Natasha Ohemeng-Kumi [32, 112], John Ramble [32, 112], Jasveen Sehmi [32, 112], Rebecca Williams [33], Wendy Chatterton [34], Monika Pusok [34], William Everson [37], Anibolina Castigador [44], Emily Macnaughton [44], Kate El Bouzidi [45], Temi Lampejo [45], Malur Sudhanva [45], Cassie Breen [47], Graciela Sluga [48], Shazaad SY Ahmad [49, 70], Ryan P George [49], Nicholas W Machin [49, 70], Debbie Binns [50], Victoria James [50], Rachel Blacow [55], Lindsay Coupland [58], Louise Smith [59], Edward Barton [60], Debra Padgett [60], Garren Scott [60], Aidan Cross [61], Mariyam Mirfenderesky [61], Jane Greenaway [62], Kevin Cole [64], Phillip Clarke [67], Nichola Duckworth [67], Sarah Walsh [67], Kelly Bicknell [68], Robert Impey [68], Sarah Wyllie [68], Richard Hopes [70], Chloe Bishop [72], Vicki Chalker [72], Ian Harrison [72], Laura Gifford [74], Zoltan Molnar [77], Cressida Auckland [79], Cariad Evans [85, 109], Kate Johnson [85, 109], David G Partridge [85, 109], Mohammad Raza [85, 109], Paul Baker [86], Stephen Bonner [86], Sarah Essex [86], Leanne J Murray [86], Andrew I Lawton [87], Shirelle Burton-Fanning [89], Brendan AI Payne [89], Sheila Waugh [89], Andrea N Gomes [91], Maimuna Kimuli [91], Darren R Murray [91], Paula Ashfield [92], Donald Dobie [92], Fiona Ashford [93], Angus Best [93], Liam Crawford [93], Nicola Cumley [93], Megan Mayhew [93], Oliver Megram [93], Jeremy Mirza [93], Emma Moles-Garcia [93], Benita Percival [93], Megan Driscoll [96], Leah Ensell [96], Helen L Lowe [96], Laurentiu Maftei [96], Matteo Mondani [96], Nicola J Chaloner [99], Benjamin J Cogger [99], Lisa J Easton [99], Hannah Huckson [99], Jonathan Lewis [99], Sarah Lowdon [99], Cassandra S Malone [99], Florence Munemo [99], Manasa Mutingwende [99], Roberto Nicodemi [99], Olga Podplomyk [99], Thomas Somassa [99], Andrew Beggs [100],

Alex Richter [100], Claire Cormie [102], Joana Dias [102], Sally Forrest [102], Ellen E Higginson [102], Mailis Maes [102], Jamie Young [102], Rose K Davidson [103], Kathryn A Jackson [107], Lance Turtle [107], Alexander J Keeley [109], Jonathan Ball [113], Timothy Byaruhanga [113], Joseph G Chappell [113], Jayasree Dey [113], Jack D Hill [113], Emily J Park [113], Arezou Fanaie [114], Rachel A Hilson [114], Geraldine Yaze [114] and Stephanie Lo [116]

Sequencing and analysis:
Safiah Afifi [10], Robert Beer [10], Joshua Maksimovic [10], Kathryn McCluggage [10], Karla Spellman [10], Catherine Bresner [11], William Fuller [11], Angela Marchbank [11], Trudy Workman [11], Ekaterina Shelest [13, 81], Johnny Debebe [18], Fei Sang [18], Marina Escalera Zamudio [23], Sarah Francois [23], Bernardo Gutierrez [23], Tetyana I Vasylyeva [23], Flavia Flaviani [31], Manon Ragonnet-Cronin [39], Katherine L Smollett [42], Alice Broos [53], Daniel Mair [53], Jenna Nichols [53], Kyriaki Nomikou [53], Lily Tong [53], Ioulia Tsatsani [53], Sarah O'Brien [54], Steven Rushton [54], Roy Sanderson [54], Jon Perkins [55], Seb Cotton [56], Abbie Gallagher [56], Elias Allara [70, 102], Clare Pearson [70, 102], David Bibby [72], Gavin Dabrera [72], Nicholas Ellaby [72], Eileen Gallagher [72], Jonathan Hubb [72], Angie Lackenby [72], David Lee [72], Nikos Manesis [72], Tamyo Mbisa [72], Steven Platt [72], Katherine A Twohig [72], Mari Morgan [74], Alp Aydin [75], David J Baker [75], Ebenezer Foster-Nyarko [75], Sophie J Prosolek [75], Steven Rudder [75], Chris Baxter [77], Sílvia F Carvalho [77], Deborah Lavin [77], Arun Mariappan [77], Clara Radulescu [77], Aditi Singh [77], Miao Tang [77], Helen Morcrette [79], Nadua Bayzid [96], Marius Cotic [96], Carlos E Balcazar [104], Michael D Gallagher [104], Daniel Maloney [104], Thomas D Stanton [104], Kathleen A Williamson [104], Robin Manley [105], Michelle L Michelsen [105], Christine M Sambles [105], David J Studholme [105], Joanna Warwick-Dugdale [105], Richard Eccles [107], Matthew Gemmell [107], Richard Gregory [107], Margaret Hughes [107], Charlotte Nelson [107], Lucille Rainbow [107], Edith E Vamos [107], Hermione J Webster [107], Mark Whitehead [107], Claudia Wierzbicki [107], Adrienn Angyal [109], Luke R Green [109], Max Whiteley [109], Emma Betteridge [116], Iraad F Bronner [116], Ben W Farr [116], Scott Goodwin [116], Stefanie V Lensing [116], Shane A McCarthy [116, 102], Michael A Quail [116], Diana Rajan [116], Nicholas M Redshaw [116], Carol Scott [116], Lesley Shirley [116] and Scott AJ Thurston [116]

Software and analysis tools:
Will Rowe [43], Amy Gaskin [74], Thanh Le-Viet [75], James Bonfield [116], Jennifier Liddle [116] and Andrew Whitwham [116]

1 Barking, Havering and Redbridge University Hospitals NHS Trust, 2 Barts Health NHS Trust, 3 Belfast Health & Social Care Trust, 4 Betsi Cadwaladr University Health Board, 5 Big Data Institute, Nuffield Department of Medicine, University of Oxford, 6 Blackpool Teaching Hospitals NHS Foundation Trust, 7 Bournemouth University, 8 Cambridge Stem Cell Institute, University of Cambridge, 9 Cambridge University Hospitals NHS Foundation Trust, 10 Cardiff and Vale University Health Board, 11 Cardiff University, 12 Centre for Clinical Infection and Diagnostics Research, Department of Infectious Diseases, Guy's and St Thomas' NHS Foundation Trust, 13 Centre for Enzyme Innovation, University of Portsmouth, 14 Centre for Genomic Pathogen Surveillance, University of Oxford, 15 Clinical Microbiology Department, Queens Medical Centre, Nottingham University Hospitals NHS Trust, 16 Clinical Microbiology, University Hospitals of Leicester NHS Trust, 17 County Durham and Darlington NHS Foundation Trust, 18 Deep Seq, School of Life Sciences, Queens Medical Centre, University of Nottingham, 19 Department of

Infectious Diseases and Microbiology, Cambridge University Hospitals NHS Foundation Trust, 20 Department of Medicine, University of Cambridge, 21 Department of Microbiology, Kettering General Hospital, 22 Department of Microbiology, South West London Pathology, 23 Department of Zoology, University of Oxford, 24 Division of Virology, Department of Pathology, University of Cambridge, 25 East Kent Hospitals University NHS Foundation Trust, 26 East Suffolk and North Essex NHS Foundation Trust, 27 East Sussex Healthcare NHS Trust, 28 Gateshead Health NHS Foundation Trust, 29 Great Ormond Street Hospital for Children NHS Foundation Trust, 30 Great Ormond Street Institute of Child Health (GOS ICH), University College London (UCL), 31 Guy's and St. Thomas' Biomedical Research Centre, 32 Guy's and St. Thomas' NHS Foundation Trust, 33 Hampshire Hospitals NHS Foundation Trust, 34 Health Services Laboratories, 35 Heartlands Hospital, Birmingham, 36 Hub for Biotechnology in the Built Environment, Northumbria University, 37 Hull University Teaching Hospitals NHS Trust, 38 Imperial College Healthcare NHS Trust, 39 Imperial College London, 40 Infection Care Group, St George's University Hospitals NHS Foundation Trust, 41 Institute for Infection and Immunity, St George's University of London, 42 Institute of Biodiversity, Animal Health & Comparative Medicine, 43 Institute of Microbiology and Infection, University of Birmingham, 44 Isle of Wight NHS Trust, 45 King's College Hospital NHS Foundation Trust, 46 King's College London, 47 Liverpool Clinical Laboratories, 48 Maidstone and Tunbridge Wells NHS Trust, 49 Manchester University NHS Foundation Trust, 50 Microbiology Department, Buckinghamshire Healthcare NHS Trust, 51 Microbiology, Royal Oldham Hospital, 52 MRC Biostatistics Unit, University of Cambridge, 53 MRC-University of Glasgow Centre for Virus Research, 54 Newcastle University, 55 NHS Greater Glasgow and Clyde, 56 NHS Lothian, 57 NIHR Health Protection Research Unit in HCAI and AMR, Imperial College London, 58 Norfolk and Norwich University Hospitals NHS Foundation Trust, 59 Norfolk County Council, 60 North Cumbria Integrated Care NHS Foundation Trust, 61 North Middlesex University Hospital NHS Trust, 62 North Tees and Hartlepool NHS Foundation Trust, 63 North West London Pathology, 64 Northumbria Healthcare NHS Foundation Trust, 65 Northumbria University, 66 NU-OMICS, Northumbria University, 67 Path Links, Northern Lincolnshire and Goole NHS Foundation Trust, 68 Portsmouth Hospitals University NHS Trust, 69 Public Health Agency, Northern Ireland, 70 Public Health England, 71 Public Health England, Cambridge, 72 Public Health England, Colindale, 73 Public Health Scotland, 74 Public Health Wales, 75 Quadram Institute Bioscience, 76 Queen Elizabeth Hospital, Birmingham, 77 Queen's University Belfast, 78 Royal Brompton and Harefield Hospitals, 79 Royal Devon and Exeter NHS Foundation Trust, 80 Royal Free London NHS Foundation Trust, 81 School of Biological Sciences, University of Portsmouth, 82 School of Health Sciences, University of Southampton, 83 School of Medicine, University of Southampton, 84 School of Pharmacy & Biomedical Sciences, University of Portsmouth, 85 Sheffield Teaching Hospitals NHS Foundation Trust, 86 South Tees Hospitals NHS Foundation Trust, 87 Southwest Pathology Services, 88 Swansea University, 89 The Newcastle upon Tyne Hospitals NHS Foundation Trust, 90 The Queen Elizabeth Hospital King's Lynn NHS Foundation Trust, 91 The Royal Marsden NHS Foundation Trust, 92 The Royal Wolverhampton NHS Trust, 93 Turnkey Laboratory, University of Birmingham, 94 University College London Division of Infection and Immunity, 95 University College London Hospital Advanced Pathogen Diagnostics Unit, 96 University College London Hospitals NHS Foundation Trust, 97 University Hospital Southampton NHS Foundation Trust, 98 University Hospitals Dorset NHS Foundation Trust, 99 University Hospitals Sussex NHS Foundation Trust, 100 University of Birmingham, 101

University of Brighton, 102 University of Cambridge, 103 University of East Anglia, 104 University of Edinburgh, 105 University of Exeter, 106 University of Kent, 107 University of Liverpool, 108 University of Oxford, 109 University of Sheffield, 110 University of Southampton, 111 University of St Andrews, 112 Viapath, Guy's and St Thomas' NHS Foundation Trust, and King's College Hospital NHS Foundation Trust, 113 Virology, School of Life Sciences, Queens Medical Centre, University of Nottingham, 114 Watford General Hospital, 115 Wellcome Centre for Human Genetics, Nuffield Department of Medicine, University of Oxford, 116 Wellcome Sanger Institute, 117 West of Scotland Specialist Virology Centre, NHS Greater Glasgow and Clyde, 118 Whittington Health NHS Trust
